# The role of Theory of Mind in the transition towards suicidal attempts in youth NSSI: an exploratory pilot study

**DOI:** 10.3389/fpsyt.2024.1403038

**Published:** 2024-05-30

**Authors:** Laura Orsolini, Diana Corona, Alessandro Leonardo Cervelli, Enrico Ribuoli, Giulio Longo, Umberto Volpe

**Affiliations:** Unit of Clinical Psychiatry, Department of Neurosciences/Department of Experimental and Clinical Neurosciences (DIMSC), Polytechnic University of Marche, Ancona, Italy

**Keywords:** non-suicidal self-injury, NSSI, suicide attempts, suicidality, adolescent, youth mental health, youths

## Abstract

Non-suicidal self-injury (NSSI) can both precede and co-occur with suicidal attempts (SA). Underlying mechanisms/factors leading to the transition to SA in NSSI youths have been proposed (including the role of social cognition), despite they should be yet confirmed. Therefore, the study aims at exploring the role of the Theory of Mind in the differentiation of a sample of NSSI youngsters (aged 15-24) according to the presence of SA. We divided the sample into 4 groups using the Deliberate Self Harm Inventory (DSHI) and Columbia Suicide Severity Rating Scale (C-SSRS): control group (notNSSInotSA), NSSI without SA (NSSInotSA), NSSI with SA (NSSIplusSA), and SA without NSSI (SAonly). NSSIplusSA patients displayed higher Reading the Mind in the Eyes Test (RMET) scores (indicative of ToM abilities) than both the NSSInotSA (p=0.0016) and SAonly groups (p=0.0198), while SAonly patients showed lower RMET scores compared to the control group (p=0.0214). Multiple regression models used to differentiate NSSInotSA and NSSIplusSA found a significant association between RMET and LOSCS-CSC (Level Of Self-Criticism Scale-Comparative Self-Criticism) (pC=0.0802, pD=0.0016, pG=0.0053). Our findings supported the hypothesis that a hypertrophic affective ToM may possibly be associated with the occurrence of SA in youth NSSI. Further larger and longitudinal studies should confirm these preliminary findings, by exploring all social cognition dimensions.

## 1 Introduction

Non-suicidal self-injury (NSSI) consists in the use of non-lethal, self-aimed, deliberate behaviours leading to the destruction of one’s own body tissue, in the absence of the aim to end one’s life (1). It commonly manifests during early adolescence, with an average age of onset between 13 and 16-years-old (2), an age characterised by drastic changes and stressors that can notably facilitate the emergence of personal vulnerabilities and maladaptive strategies. Recent meta-analytic work stated how the occurrence of NSSI behaviour during development shows an initial increase in correspondence of early adolescence, followed by a peak and a subsequent decline (3). Reported data sums up to a relatively high prevalence in this population, estimated to be 16% (4), and, as such, NSSI is identified as a major public health concern (5, 6), even more so as it associates with different negative outcomes, including suicidal behaviours (7). Suicidal behaviours are defined as nonfatal suicidal thoughts and behaviours and classified as a) suicide ideation, the presence of thoughts of ending one’s life; b) suicide plans, the formulation of a specific method to do so; c) suicide attempts (SA), which refer to potentially self-injurious behaviours in which there is at least some intent, overt or inferred, to die (8). These behaviours are well-known harbingers of suicide death in youths, that represent the fourth leading cause of death among 15-19 year olds worldwide (9, 10), with reported global suicide rate amounting to 3.8 per 100,000 people among adolescents (11).

Overall, NSSI can co-occur and precede SA. In fact, 70% of youths with NSSI reported a positive history of at least one SA and a SA risk 3-fold higher than youths without NSSI (12). Therefore, NSSI has been identified as one of the strongest predictors of SA by both cross-sectional and longitudinal studies (13, 14). Despite vast literature on the matter, the etiopathogenesis underpinning this association has yet to be clarified (15, 16). Some neurobiological studies highlighted the role of emotional intelligence and emotional dysregulation (17). Other works have theorised possible facilitating mechanisms underlying both phenomena. Since NSSI was associated with higher lethality of suicide attempts (18), it was theorised that pain desensitisation induced by NSSI could facilitate the enactment of suicidal thoughts (19, 20), leading to an acquired capability to commit SA (21, 22). Other studies hypothesised a psychopathological continuum of self-injurious behaviours with NSSI escalating in SA, particularly when associated with high psychological distress (23, 24). Finally, other authors proposed the role of shared risk factors and, particularly, the identification of specific psycho-social vulnerability domains shared by NSSI and SA (25–27).

In fact, literature seems to point out several areas that could underlie both phenomena, including depressive symptomatology (28–30), dissociative symptoms (31), impulsiveness (31–34), emotion identification (35, 36), expression (37, 38) and dysregulation (39–41), aggressivity and anger pervasiveness (42), feelings of worthlessness (43), and social reactivity (e.g., sensitivity to interpersonal rejection) (44, 45). Despite that, data on possible differences between NSSI and SA relative to these factors is relatively scarce, particularly in youths.

Therefore, our study aimed to investigate the role of a set of psychopathological dimensions in distinguishing between NSSI youths with or without a lifetime history of SA. In particular, our primary aim was to investigate whether current social cognition could discriminate NSSI youths with or without SA, as previous studies suggested its impairment in suicidal attempters (46). Social cognition includes empathy (e.g., the ability to understand the mental states of others and responding to them with affective mobilisation) and the theory of the Mind (ToM), i.e. the ability to infer the emotional states of others based on social cues (47, 48). We specifically explored the affective component of ToM, referring to the understanding of feelings and emotions of others (46). We presume it has a role in suicidality shift in NSSI youths, as it can influence youth engagement in prosocial behaviour, and the development of effective interpersonal communication and interpersonal reactivity/vulnerability. Secondary outcomes investigated whether specific dysfunctional coping patterns, such as emotional dysregulation, anger rumination and self-criticism, could contribute to the development of SA in youth NSSI. Exploratory outcomes included the role of dissociation and alexithymia as precipitating and/or mediating agents for SA among at-risk NSSI youths. The final goal was to preliminarily explore in a sample of youths which variables could help to clinically stratify NSSI youths at-risk for SA, through a pilot study carried out in a real-world setting, ultimately leading to target-specific preventive and treatment programs.

## 2 Method

### 2.1 Study design and selection of participants

A retrospective chart-review study was carried out by recruiting all adolescent and young inpatients hospitalised at our Transition Psychiatry Inpatient Service, and outpatients afferent to our Transition Psychiatry Outpatient Service at the Unit of Clinical Psychiatry, University Hospital of Marche, Polytechnic University of Marche, Ancona (Italy), during the timeframe September 2020 to December 2023. A total of 72 patients were involved in this study. Written informed consent was obtained from the patients or their parents (when aged less than 18-year-old) after they were informed about the purpose of the study. Patients were retrospectively included in the study if they met the following inclusion criteria: a) aged 15-24; b) education level not lower than elementary school, to ensure ability to read and correctly interpret the proposed scales; c) absence of active psychotic symptomatology at the evaluation; d) signed informed consent for collecting and analysing clinical data for research purpose, collected during baseline assessment. Participants were excluded if they met one or more of the following: a) intellectual disability or cognitive impairment; b) diagnosis of organic mental disorder according to the DSM-5 criteria (49); c) being either under the influence of substances and/or alcohol at the moment of the evaluation; d) incomplete filled out questionnaires; e) linguistic difficulties (i.e., not Italian speaker or foreign without a sufficient ability to understand Italian language). Recruited patients had also the possibility to withdraw their participation without any clinical or therapeutic consequence. All procedures performed in studies involving human participants were in accordance with the ethical standards of the institutional and/or national research committee and with the 1964 Helsinki Declaration and its later amendments or comparable ethical standards. The Institutional Review Board approved our study (Prot 32/2024). This research study was conducted retrospectively from data obtained for clinical purposes.

### 2.2 Measures

An *ad hoc* case report form was specifically designed and hetero-administered by the researchers to collect sociodemographic (e.g., age, ethnicity, marital status, living status, parental marital status, employment status, education level) and clinical data (e.g., personal and family psychiatric history). History of NSSI and SA was assessed through the administration by a trained clinician of the Italian version of the Deliberate Self-Harm Inventory (50, 51) and the Columbia–Suicide Severity Rating Scale (C-SSRS) (52). The DSHI is a 17-item behaviourally based questionnaire that identifies the manifestation of self-harm without conscious suicidal intent. Respondents answer whether or not they engaged in specific acts (dichotomous answer), by providing also frequency and time of onset. DSHI displays a Cronbach’s alpha of 0.82, indicating high internal consistency. The DSHI showed adequate test-retest reliability over a period ranging from 2 to 4 weeks (50, 51). The C-SSRS is a clinician-administered questionnaire assessing suicidal risk, by evaluating both suicidal ideation and behaviour. The SA subscale is rated on a nominal scale that includes actual, aborted, and interrupted attempts, preparatory behaviours, nonsuicidal self-injurious behaviour (52).

Moreover, a set of assessment tools to investigate clinical and psychological dimensions were administered to all participants (listed below). All scales and questionnaires, even if self-administered, were compiled in the presence of healthcare personnel, in order to favour full engagement of the patients in the task.

Reading the Mind in the Eyes Test (RMET), used to assess affective ToM, is a 36- item that presents participants with photographs of a set of eyes and asks them to identify the emotion displayed from 4 choices. Total score ranges from 0 to 36, where a typical score is in the range 22-30 and < 22 indicates difficulties in emotion recognition. The validation study on the Italian version herein adopted confirmed internal consistency with a Cronbach’s α of 0.605 (53, 54).

Difficulties in Emotion Regulation Strategies (DERS) is a widely-used measure to assess difficulties in emotion regulation. It consists of 36 self-report items on a 5-point Likert scale, with responses from 1 to 5, ranging from “almost never” to “almost always”. Total scores range from 36 to 180, with higher scores suggesting greater problems with emotion regulation. The Italian version adopted in this study identifies the following subscales: Non-acceptance of emotional responses (Non-Acceptance), Difficulty engaging in distracting behaviours (Distracting), Impulse control difficulties (Impulse), Lack of emotional awareness (Awareness), Limited access to emotion regulation strategies (Strategies), Lack of emotional clarity (Clarity). The total score displays a high internal consistency (α = 0.90), as well each subscale presenting a Cronbach’s α ranging from 0.74 to 0.88 (55, 56).

Anger Rumination Scale (ARS) assesses the tendency to focus attention on angry moods, on current anger-provoking situations and recall past anger episodes. The Italian version we adopted consists of a 13-item self-report tool rated on a 4-point Likert scale ranging from 1 to 4 (“almost never” to “almost always”). Total scores range from 13 to 52, with higher scores indicating higher tendency to dwell in anger rumination. An excellent internal consistency (α = 0.93) and a 1‐month test‐retest reliability of 0.77 was reported (57, 58).

Level Of Self-Criticism Scale (LOSCS) measures two dimensions of self-criticism: Comparative Self-Criticism (CSC) and Internalised Self-Criticism (ISC). The Italian version used in our study consists of a 22-item self-report questionnaire scored over a Likert scale from 1 (“not at all”) to 7 (“very well”). Total scores range from 22 to 154, with higher scores linked to higher self-criticism. A good internal consistency was reported for both CSC (α = 0.81) and ISC (α = 0.87) (59, 60).

Dissociative Experiences Scale (DES-II) is a 28-item, self-report measure of the frequency of dissociative experiences such as derealisation, depersonalisation, absorption and amnesia. Total scores range from 0 to 100, where high levels of dissociation are indicated by scores of 30 or more. A high internal consistency (α = 0.94) was reported for the Italian version (61).

Toronto Alexithymia Scale (TAS-20) measures difficulty in identifying and describing emotions. It is a 20-item, self-administered questionnaire, scored 1 to 5, that comprises three scales: Difficulty Identifying Feelings (DIF), Difficulty Describing Feelings (DDF), and Externally Oriented Thinking (EOT). Total score ≤ 49 is negative for alexithymia, 50-60 equals to undetermined results whereas ≥ 61 indicates the presence of alexithymia. The Italian version reported a satisfactory Cronbach’s α in community (0.75) and clinical population (0.82) (62, 63).

### 2.3 Statistical analysis

Participants were divided in four groups, according to DSHI and C-SSRS: a) subjects manifesting NSSI without previous history of SA (NSSInotSA); b) subjects manifesting both phenomena (NSSIplusSA); c) subjects manifesting SA without history of NSSI (SAonly); d) subjects without a history of NSSI nor SA (notNSSInorSA, acting as clinical control group). Subjects with NSSI were identified when a yearly frequency of NSSI > 5 events/year was reported at the DSHI. Subjects with SA were identified as those whose total sum of the C-SSRS Suicidal behaviours subscale items “Total # of Attempts” and “Total # of interrupted” amounted at more than 1 attempt (Total# ≥ 1). Descriptive statistics were expressed as mean and standard deviation (SD) for the quantitative variables, after confirming normality of their distribution through Shapiro-Wilk test. Qualitative variables were presented in absolute frequency (n) and percentage (%). Association between qualitative variables and the distribution of the four groups under study were tested through χ2-tests. One-way analysis of variance (ANOVA) was performed to compare all continuous variables across the four groups and, whenever a statistically significant group effect was observed, differences between groups were further investigated through pairwise t-tests with pooled SD. P-values for pairwise t-tests were adjusted through the Benjamini & Hochberg method for p-value correction (64). Finally, quantitative variables showing group effect were tested for possible association with all others in respect to NSSI group differentiation through multiple linear regression models with two predictor variables, one of which was always the factor defining the belonging to either NSSInotSA or NSSIplusSA. All statistical analyses were performed using R Statistical Software (Version 4.3.3, R Core Team 2024).

## 3 Results

### 3.1 Socio-demographic and clinical features of the sample

All socio-demographic characteristics are summarised in **Table 1**. A total of 72 adolescents and young adults were consecutively assessed during the timeframe September 2020-December 2023. Most of the sample consisted of females (81.9%), without any significant difference across four groups (p=0.2587). The mean age was 17.7 years (SD=2.4), without significant differences across four groups (p=0.3971). χ2-test for the inpatient/outpatient categories confirmed that patients with SA were more likely to have accessed our clinic through hospitalisation rather than outpatient treatment (p=0.0019). Among the studied sample, the most represented primary diagnosis was Bipolar Disorder (26.4%), followed by Personality Disorder (22.2%) and Depressive Disorder (19,4%), with Mood Disorders comprehensively amounting to more than 45% of the entire sample.

**Table 1:** Socio-demographic features of the sample.

		nonNSSInorSA	NSSInotSA	NSSIplusSA	SAonly	TOTAL	TEST p-value
**TOTAL** Number (%)		21 (29.2%)	21 (29.2%)	20 (27.8%)	10 (13.9%)	72	
**AGE** Mean (SD)		18.29 (2.72)	17.33 (2.46)	17.25 (1.68)	18.30 (2.91)	17.72 (2.42)	0.3971
**YEARS OF EDUCATION** Mean (SD)		11.71 (2.28)	10.90 (1.55)	11.25 (2.36)	12.20 (2.10)	11.42 (2.09)	0.3673
ETHNICITY	**CAUCASIAN** Frequency (%)	20 (95.2%)	20 (95.2%)	17 (85.0%)	8 (80.0%)	65 (90.3%)	0.3545
	**AFRICAN** Frequency (%)	0 (0%)	1 (4.8%)	2 (10.0%)	0 (0%)	3 (4.2%)	
	**SOUTH-AMERICAN** Frequency (%)	0 (0%)	0 (0%)	1 (5.0%)	1 (10.0%)	2 (2.8%)	
	**ASIAN** Frequency (%)	1 (4.8%)	0 (0%)	0 (0%)	1 (10.0%)	2 (2.8%)	
GENDER	**FEMALE** Frequency (%)	15 (71.4%)	18 (85.7%)	16 (80.0%)	10 (100.0%)	59 (81.9%)	0.2587
	**MALE** Frequency (%)	6 (28.6%)	3 (14.3%)	4 (20.0%)	0 (0%)	13 (18.1%)	
OCCUPATION	**STUDENT** Frequency (%)	17 (81.0%)	19 (90.5%)	17 (85.0%)	9 (90.0%)	62 (86.1%)	0.6787
	**WORKER** Frequency (%)	1 (4.8%)	0 (0%)	2 (10.0%)	0 (0%)	3 (4.2%)	
	**UNEMPLOYED** Frequency (%)	3 (14.3%)	2 (9.5%)	1 (5.0%)	1 (10.0%)	7 (9.7%)	
LIVING STATUS	**WITH FAMILY OF ORIGIN** Frequency (%)	19 (90.5%)	18 (85.7%)	19 (95.0%)	10 (100.0%)	66 (91.7%)	0.6536
	**ALONE** Frequency (%)	2 (9.5%)	1 (4.8%)	0 (0%)	0 (0%)	3 (4.2%)	
	**WITH A PARTNER** Frequency (%)	0 (0%)	1 (4.8%)	0 (0%)	0 (0%)	1 (1.4%)	
	**FOSTER CARE** Frequency (%)	0 (0%)	1 (4.8%)	1 (5.0%)	0 (0%)	2 (2.8%)	
PARENTAL MARITAL STATUS	**LIVING TOGETHER** Frequency (%)	17 (81.0%)	14 (66.7%)	13 (65.0%)	6 (60.0%)	50 (69.4%)	0.3312
	**SEPARATED/** **DIVORCED** Frequency (%)	2 (9.5%)	6 (28.6%)	7 (35.0%)	4 (40.0%)	19 (26.4%)	
	**WIDOWED** Number (%)	2 (9.5%)	1 (4.8%)	0 (0%)	0 (0%)	3 (4.2%)	
TYPE	**INPATIENT** Number (%)	7 (33.3%)	12 (57.1%)	15 (75.0%)	10 (100.0%)	44 (61.1%)	**0.0019**
	**OUTPATIENT** Number (%)	14 (66.7%)	9 (42.9%)	5 (25.0%)	0 (0%)	28 (38.9%)	
DIAGNOSIS	**NONE** Number (%)	1 (4.8%)	0 (0%)	1 (5.0%)	0 (0%)	2 (2.8%)	**0.0043**
	**PSYCHOTIC** Number (%)	0 (0%)	0 (0%)	1 (5.0%)	0 (0%)	1 (1.4%)	
	**BIPOLAR** Number (%)	2 (9.5%)	6 (28.6%)	8 (40.0%)	3 (30.0%)	19 (26.4%)	
	**DEPRESSIVE** Number (%)	6 (28.6%)	1 (4.8%)	5 (25.0%)	2 (20.0%)	14 (19.4%)	
	**ANXIETY** Number (%)	2 (9.5%)	1 (4.8%)	0 (0%)	0 (0%)	3 (4.2%)	
	**OCD** Number (%)	6 (28.6%)	0 (0%)	0 (0%)	0 (0%)	6 (8.3%)	
	**PTSD** Number (%)	2 (9.5%)	1 (4.8%)	1 (5.0%)	2 (20.0%)	6 (8.3%)	
	**EATING DISORDER** Number (%)	0 (0%)	5 (23.8%)	0 (0%)	0 (0%)	5 (6.9%)	
	**PERSONALITY DISORDER** Number (%)	2 (9.5%)	7 (33.3%)	4 (20.0%)	3 (30.0%)	16 (22.2%)	
FAMILY HISTORY OF PSYCHIATRIC DISORDER	**NONE** Number (%)	9 (42.9%)	10 (47.6%)	11 (55.5%)	5 (50.0%)	35 (48.6%)	0.5788
	**PSYCHOTIC** Number (%)	0 (0%)	0 (0%)	2 (10.0%)	0 (0%)	2 (2.8%)	
	**BIPOLAR** Number (%)	2 (9.5%)	0 (0%)	1 (5.0%)	0 (0%)	3 (4.2%)	
	**DEPRESSIVE** Number (%)	1 (4.8%)	5 (23.8%)	2 (10.0%)	2 (20.0%)	10 (13.9%)	
	**ANXIETY** Number (%)	3 (14.3%)	1 (4.8%)	0 (0%)	1 (10.0%)	5 (6.9%)	
	**OCD** Number (%)	1 (4.8%)	0 (0%)	0 (0%)	0 (0%)	1 (1.4%)	
	**PTSD** Number (%)	0 (0%)	1 (4.8%)	0 (0%)	0 (0%)	1 (1.4%)	
	**EATING DISORDER** Number (%)	2 (9.5%)	1 (4.8%)	0 (0%)	0 (0%)	3 (4.2%)	
	**SUBSTANCE USE DISORDER** Number (%)	0 (0%)	1 (4.8%)	2 (10.0%)	1 (10.0%)	4 (5.6%)	
	**PERSONALITY DISORDER** Number (%)	3 (14.3%)	2 (9.5%)	2 (10.0%)	1 (10.0%)	8 (11.1%)	

In bold significant p-values.

The χ2-test test revealed significant differences among the 4 groups regarding frequencies of primary diagnosis (p=0.0043). In particular, NSSInotSA patients showed a higher-than-expected frequency of Eating Disorder diagnosis than all other groups, whereas Depressive Disorder was underrepresented for this group of patients. Moreover, the notNSSInorSA subjects were more likely to be diagnosed with OCD, and less likely to present Bipolar Disorder than all others. According to DSHI, the NSSInotSA group significantly showed an earlier age of appearance of self-harming behaviours compared to NSSIplusSA (p=0.0275).

### 3.2 Psychopathological features of participants

ANOVA revealed a group effect for multiple of the analysed scales and subscales, as reported in **Table 2**. Pairwise t-tests were thus performed to verify statistical differences among pairs of the groups under study (**Table 3**).

**Table 2:** Psychometric features of the sample and across all four groups.

VARIABLE		nonNSSInorSA	NSSInotSA	NSSIplusSA	SAonly	Total	ANOVA p-value
**RMET**	Mean	23.62	22.43	25.05	20.60	23.25	**0.0016**
	*SD*	*3.37*	*2.66*	*3.14*	*2.46*	*3.27*	
**LOSCS-ISC**	Mean	39.67	56.76	49.00	52.70	49.06	**0.0008**
	*SD*	*14.03*	*10.47*	*14.35*	*13.22*	*14.46*	
**LOSCS-CSC**	Mean	44.48	62.52	53.70	50.10	53.08	**0.0005**
	*SD*	*14.53*	*8.32*	*15.53*	*13.30*	*14.68*	
**LOSCS**	Mean	84.14	117.86	104.05	102.80	102.10	**0.0004**
	*SD*	*27.40*	*17.34*	*26.77*	*24.06*	*27.04*	
**ARS**	Mean	27.67	38.86	34.40	34.80	33.79	**0.0018**
	*SD*	*8.81*	*7.30*	*9.41*	*11.39*	*9.79*	
**DES-II**	Mean	23.57	43.76	36.30	47.70	36.35	**0.0053**
	*SD*	*17.62*	*16.97*	*21.50*	*29.81*	*22.08*	
**TAS-20 - DIF**	Mean	21.76	23.33	25.60	21.40	23.24	0.1670
	*SD*	*6.61*	*6.51*	*4.32*	*6.79*	*6.15*	
**TAS-20 - DDF**	Mean	16.48	17.48	18.20	18.80	17.57	0.3035
	*SD*	*3.91*	*3.47*	*3.85*	*2.57*	*3.63*	
**TAS-20 - EOT**	Mean	20.43	21.67	23.05	21.70	21.69	0.3193
	*SD*	*4.06*	*4.40*	*5.13*	*3.68*	*4.46*	
**TAS-20 Total**	Mean	58.05	62.19	67.80	62.80	62.63	**0.0191**
	*SD*	*9.37*	*9.76*	*9.60*	*9.80*	*10.11*	
**DERS - Non-Acceptance**	Mean	15.86	21.33	20.60	16.60	18.88	**0.0423**
	*SD*	*6.21*	*7.09*	*7.21*	*8.28*	*7.33*	
**DERS - Distracting**	Mean	16.52	21.81	19.90	16.40	18.99	**0.0027**
	*SD*	*5.57*	*4.06*	*4.29*	*6.24*	*5.35*	
**DERS - Impulse**	Mean	15.95	22.90	22.00	17.90	19.93	**0.0075**
	*SD*	*7.42*	*6.56*	*6.42*	*8.39*	*7.53*	
**DERS - Awareness**	Mean	17.24	18.95	18.35	16.70	17.97	0.5733
	*SD*	*4.94*	*5.07*	*4.85*	*5.38*	*4.98*	
**DERS - Strategies**	Mean	25.29	29.90	30.80	26.00	28.26	0.0671
	*SD*	*6.65*	*7.00*	*7.06*	*10.68*	*7.75*	
**DERS - Clarity**	Mean	7.57	9.90	9.60	8.90	9.00	0.0784
	*SD*	*2.91*	*2.95*	*3.25*	*3.35*	*3.17*	
**DERS Total**	Mean	107.52	134.10	131.35	111.30	122.42	**0.0070**
	*SD*	*25.08*	*26.59*	*24.85*	*40.97*	*30.00*	

NSSI, non-suicidal self-injury; SA, suicidal attempts; RMET, Reading the Mind in the Eyes Test; LOSCS, Level Of Self-Criticism Scale; ISC, Internalised Self-Criticism; CSC, Comparative Self-Criticism; ARS, Anger Rumination Scale; DES-II, Dissociative Experiences Scale-II; TAS-20, Toronto Alexithymia Scale-20 items; DERS, Difficulties in Emotion Regulation Strategies. In bold significant p-values.

**Table 3:** Pairwise t-tests results showing significant differences of test results among the four groups.

	*NSSIplusSA*	*NSSIplusSA*	*NSSIplusSA*	*NSSInotSA*	*NSSInotSA*	*SAonly*
	notNSSInorSA	NSSInotSA	SAonly	notNSSInorSA	SAonly	notNSSInorSA
**RMET**	0.1568	**0.0198**	**0.0016**	0.2019	0.1568	**0.0214**
**DERS**	**0.0250**	0.7550	0.1040	**0.0190**	0.7600	0.7550
**DERS - Non-Acceptance**	0.1050	0.7850	0.2220	0.0850	0.1700	0.7850
**DERS - Distracting**	0.0634	0.2629	0.1065	**0.0053**	**0.0170**	0.9480
**DERS - Strategies**	0.1300	0.8100	0.2100	0.1500	0.8100	0.2700
**DERS - Impulse**	**0.0230**	0.6820	0.2070	**0.0130**	0.1380	0.5690
**DERS - Clarity**	0.1200	0.7500	0.6700	0.1000	0.6000	0.5300
**ARS**	0.0569	0.1744	0.9086	**0.0008**	0.1744	0.0844
**LOSCS**	**0.0315**	0.1085	0.8943	**0.0002**	0.1322	0.0977
**LOSCS-ISC**	**0.0508**	0.0924	0.4673	**0.0004**	0.4673	**0.0347**
**LOSCS-CSC**	**0.0532.**	**0.0532.**	0.4825	**0.0002**	**0.04980**	0.3241
**DES-II**	0.1032	0.2994	0.2352	**0.0097**	0.6197	**0.0097**
**TAS-20**	**0.0110**	0.1980	0.2430	0.2430	0.2430	0.2430

NSSI, non-suicidal self-injury; SA, suicidal attempts; RMET, Reading the Mind in the Eyes Test; DERS, Difficulties in Emotion Regulation Strategies; ARS, Anger Rumination Scale; LOSCS, Level Of Self-Criticism Scale; ISC, Internalised Self-Criticism; CSC, Comparative Self-Criticism; DES-II, Dissociative Experiences Scale-II; TAS-20, Toronto Alexithymia Scale-20 items. In bold significant p-values.

Interestingly, NSSIplusSA patients displayed higher RMET scores than both the NSSInotSA and SAonly groups (respectively, p=0.0016 and p=0.0198), while SAonly patients showed lower RMET scores compared to the control group (p=0.0214).

DERS total scores were significantly higher in both NSSInotSA (p=0.0190) and NSSIplusSA (p=0.0250) compared to the control group, with a similar trend for the DERS Impulse subscale (p=0.0130 and p=0.0230 respectively). The DERS Distracting subscale showed significantly higher scores only in the NSSInonSA group compared to the control group (p=0.0053) and SAonly (p=0.0170).

ARS scores reported a significant difference between NSSInotSA and nonNSSInorSA (p=0.0008), with the former showing higher scores.

A similar trend emerged regarding the CSC subscale of LOSCS, with the NSSInotSA group presenting higher scores compared to notNSSInorSA (p=0.0002) and SAonly (p=0.0498). Interestingly, the p-value between the two NSSI groups for this subscale is barely above statistical significance (p=0.0532). The LOSCS scale itself evidenced higher values for both NSSI groups in respect to the control one (p=0.0002 and p=0.0315), whereas the ISC subscale reported all clinical groups with higher scores than the controls (p=0.0004 for NSSInonSA, p=0.0508 for NSSIplusSA, p=0.0347 for SAonly).

Regarding exploratory variables, significantly higher DES-II scores were found in both NSSInotSA (p=0.0097) and SAonly groups (p=0.0097), compared to the control group. Higher TAS-20 total scores were observed in NSSIplusSA compared to the control group (p=0.0110).

Multiple regression models were run to differentiate NSSInotSA and NSSIplusSA considering multiple variables (**Table 4**). Those that had a significant general p-value (pG, thus being good representation of the data), as well as statistically significant p-values for association between the two variables taken into account (pA) and for the differentiation between the NSSInonSA and NSSIplusSA groups (pD) are the following: a) LOSCS - CSC associated with TAS-20//DDF (pC=0.0138, pD=0.0105, pG=0.0043); b) LOSCS - CSC associated with DERS (pC=0.0004, pD=0.0188, pG=0.0002); c) TAS-20 associated with DERS (pC=2^-5^, pD=0.0137, pG=2^-5^); d) RMET associated with LOSCS - CSC (pC=0.0802, pD=0.0016, pG=0.0053).

**Table 4:** Multivariate Regression models statistically significative for differentiation between NSSInonSA and NSSIplusSA.

	Variables	Estimate	SE	t	F	p
LOSCS-CSC	Intercept	39.7114	9.1890	4.3220		**0.0001**
	TAS-20-DDF	1.3053	0.5056	2.5820		**0.0138**
	Groups	-9.7686	3.6293	-2.6920		**0.0105**
	Model				6.318	**0.0043**
LOSCS-CSC	intercept	28.0051	9.1147	3.0730		**0.0039**
	DERS	0.25742	0.0658	3.9150		**0.0004**
	Groups	-8.1171	3.3096	- 2.4530		**0.0189**
	Model				11.2300	**0.0002**
TAS-20	intercept	31.1238	6.6572	4.6750		**3.64^-5^**
	DERS	0.2317	0.0480	4.8240		**2.3^-5^**
	Groups	6.24553	2.4173	2.5840		**0.0137**
	Model				14.3400	**2.23^-5^**
RMET	intercept	18.3244	2.3651	7.748		**2.45^-9^**
	LOSCS-CSC	0.0656	0.0365	1.7970		0.0802
	Groups	3.200	0.9384	3.411		**0.0016**
	Model				6.0380	**0.0053**

NSSI, non-suicidal self-injury; SA, suicidal attempts; RMET, Reading the Mind in the Eyes Test; DERS, Difficulties in Emotion Regulation Strategies; LOSCS, Level Of Self-Criticism Scale; CSC, Comparative Self-Criticism; DES-II, Dissociative Experiences Scale-II; TAS-20, Toronto Alexithymia Scale-20 items. In bold significant p-values.

## 4 Discussion

The current study aims to identify a set of psychopathological dimensions between young subjects who manifest only NSSI versus those who display both NSSI and SA, to investigate which vulnerability factors could help stratifying the population of NSSI youths with respect to the risk of presentation of suicidal acts. Our primary objective was to investigate the association between the affective component of ToM and suicidality in a sample presenting NSSI or not. Our findings revealed that NSSIplusSA patients displayed statistically significant higher RMET scores compared to both the NSSInotSA and SAonly groups, while the SAonly group displayed lower scores when compared to the control group. These preliminary findings could potentially suggest that a higher affective ToM may share a distinct, significant relation with suicidality within youth NSSI. Indeed, one could argue that ToM could display different patterns across the lifespan (65) and even more so during adolescence: core features of ToM continue to develop as youngsters are faced with increasingly complex social situations amidst their brain development. Thus, in this paper we hypothesise that a hypertrophic ToM should be further explored as an age-specific marker of suicidality shift within NSSI youths: those with an hypermentalising asset tend to over-interpret information from their social environment about others’ mental states (66, 67). A higher ToM could be maladaptive for interpersonal functioning as it may lead NSSI youths to potentially mistakenly interpret rejection, abandonment or criticism, exacerbating beliefs of burdensomeness and/or lack of connectedness. This could contribute to excessive interpersonal reactivity and social distress, which in turn could determine the transition to suicidal acts. Our findings were also supported by a previous study (68). Interestingly, lower scores at RMET seem to characterise the SAonly group in our sample. This data is coherent with previous literature (36) that suggested how inaccurate mentalising patterns such as lack in others’ emotion recognition seems to be associated with suicidal behaviours. This possibly suggests the presence of different triggering mechanisms and/or underpinned afflictions determining SA in youths with or without NSSI.

A recent meta-analytic work comparing subjects with eating disorders (ED) and NSSI with both a clinical and a healthy control group, found a higher NSSI prevalence in ED subjects, without identifying any significant group-differences on SA (69). Despite our small sample, our findings also partially confirmed this meta-analysis, even though we found a higher comorbid ED diagnosis only in NSSInotSA but not in the NSSIplusSA group. Our results could suggest the possible presence of different subtypes of NSSInjurers, where self-harm as whole could hold a separate meaning and as such it could imply a different likelihood of SA co-occurrence or development. Indeed, these findings should need further replication studies.

Regarding the potential discrimination through dysfunctional coping patterns between the two NSSI groups, our findings did not find any relevant differences in emotional dysregulation dimension. Both NSSInotSA and NSSIplusSA showed significantly higher DERS scores compared to the control group, which is consistent with previous published literature. An association between NSSI and emotion regulation difficulties has been clearly confirmed (40), with NSSI being historically identified as a possible maladaptive strategy to modulate intense emotional reactions (70). Studies on SA and emotion dysregulation, instead, showed contrasting findings (41, 71, 72). Similarly, significant differences regarding the anger-type rumination dimension were not observed, as it resulted significantly higher in all three clinical groups. These findings are consistent with previous literature which supported the presence of a predominant ruminative thought pattern within both NSSI and SA, with self-injury acting as a maladaptive strategy to discontinue highly intensive ruminative cycles (73, 74), particularly in more potentially harmful or dreadful SA (42, 75, 76). Furthermore, interesting findings were observed in the self-criticism dimension, which could partially be associated with those derived by our primary outcome. Although our results observed significantly higher LOSCS scores for both NSSI groups compared to the control group, when we investigated the CSC subscale we found significantly higher scores within NSSInotSA and a subthreshold trend discriminating between two NSSI groups is observed. Indeed, aberrant self-criticism has been described as a facilitator for the development of NSSI and SA (15, 43) as NSSI may represent a sort of self-punishment in response to worthlessness ideation (77, 78), whereas specific types of self-criticism, such as the feeling of an inadequate self with tendency to perfectionism, have been found to increase likelihood of suicidality, both in adult and adolescent samples.

Furthermore, findings relative to exploratory variables warrant for further investigation of dissociative symptomatology. Previous literature already documented the role of dissociative symptomatology in youths with history of NSSI and SA (31, 79), but no discrimination between the NSSI groups (NSSInotSA and NSSIplusSA) was ever suggested. It was proposed that NSSI could act as an “anti-dissociative” (80), while other researchers suggested the presence of a dissociative subtype of NSSI in which self-harm could have a “pro-dissociative function”, as physical pain could facilitate emotional and mental distress anaesthesia (81). Interestingly, the latter has been associated with a shorter shift towards SA (82) and dissociation was suggested as SA facilitator, as it could favour numbness to physical pain and disconnection from one’s body (83), a theory that has been also explored by a study using virtual reality (84). Our findings described significant higher DES-II scores in NSSInotSA and SAonly groups compared to the control group, whereas the NSSIplusSA group showed lower scores, hence suggesting a potential ‘protective’ role of dissociation regarding the presentation of SA among NSSI individuals exclusively. Thus, we suggest that a subtype of NSSI youths at higher risk of suicidality could be identified depending on dissociative dimension.

Overall, despite our exploratory pilot study shedding light on interesting findings, several limitations to the current work should be properly addressed. Firstly, the cross-sectional design precludes causal inferences between ToM and suicidality risk in youth NSSI. Secondly, the relatively small sample prevented us from comparing subgroups and may have invalidated statistical significance where we clearly found a subthreshold trend discriminating between two NSSI groups. Numerosity is particularly relevant in this study as the sample has been divided in 4 groups, thus increasing it could help clarify some borderline situations and reduce statistical error. Moreover, our study did not investigate possible ToM variations determined by age, symptomatology, nor severity of illness. Ultimately, we relied on RMET to preliminarily explore potential variations of ToM: psychometric properties of the test have been recently debated (85), despite the Italian validation study confirming its validity (54). Hence, these limitations contribute to the aforementioned preliminary and pilot nature of the current study, which should be further strengthened by recruiting a larger sample size, including adult subjects, with longitudinal design and a full set of rigorous social cognition assessment tools.

Overall, current findings provide significant implications for future research directions, as well as for timely and target-specific clinical intervention for suicidality risk in youth NSSI. Our primary outcome suggested a role of affective component of ToM in suicidality enactment among NSSI youths. This should be extensively investigated in both clinical and neuroimaging studies, in addition to emotional intelligence, empathy and cognitive components of ToM, to define their role in interpersonal hyper-reactivity which could underpin higher risk to act suicide. Finally, interventional studies should also evaluate which social-cognitive interventions (such as mentalisation-based therapies, cognitive behavioural therapy, etc.) (86, 87) could effectively address this hypermentalising dimension in at-risk youths, possibly aiming at the reduction of social sensitivity-induced distress and implementation of more functional interpersonal strategies.

## Data availability statement

The datasets presented in this article are not readily available because of identifiable participants’ data. Requests to access the datasets should be directed to l.orsolini@staff.univpm.it.

## Ethics statement

The studies involving humans were approved by Local Institutional Review Board. The studies were conducted in accordance with the local legislation and institutional requirements. Written informed consent for participation in this study was provided by the participants’ legal guardians/next of kin.

## Author contributions

LO: Writing – review & editing, Writing – original draft, Visualization, Validation, Supervision, Resources, Methodology, Conceptualization. DC: Writing – original draft, Investigation, Formal analysis, Data curation. AC: Writing – review & editing, Investigation, Data curation. ER: Writing – review & editing, Supervision, Investigation, Data curation. GL: Resources, Writing – review & editing, Visualization. UV: Writing – review & editing, Visualization, Validation, Supervision.
